# Supplementary material for: Determinants of soil-transmitted helminth infections among pre-school-aged children in Gamo Gofa zone, Southern Ethiopia: A case-control study
Source: PLoS One. 2020 Dec 11;15(12):e0243836. doi: 10.1371/journal.pone.0243836 (PMC7732061; doi:10.1371/journal.pone.0243836)
Supplement: S1 Questionnaire — (DOCX) [file pone.0243836.s001.docx]

**S1 Questionnaire**

**Part one: Socio-demographic characteristics of caregivers and economic characteristics of households.**

***Kebele*__________________________ House number______________ Child ID______**

| **Serial number** | | **Questions** | **Option** | |
| --- | --- | --- | --- | --- |
| **Socio-demographic characteristics caregivers** | | | | |
| 101 | | How old are you? | ______ (years) | |
| 102 | | Where is your place of residence? | 1.Urban 2.Rural | |
| 103 | | What is your current marital status? | 1.Married 2.Unmarried/single  3. Separated 4. Windowed  5.Others(specify) _________ | |
| 104 | | What is the highest educational level you achieved? | 1.Can’t read and write  2.Can read write  3.Grade1-8  4.Grade 9-12  5.College and above | |
| 105 | | What is your occupation? | 1.Farmer  2.Employed (government)  3.Business (Self-employed)  4. Housewife  5.Unemployed  6.Other (specify) _________ | |
| 106 | | How many individuals live in the household? | ______ (in number) | |
| **Economic characteristics of households (1. Yes 0. No)** | | | | |
| 107 | Is there electricity in home? | | 1. 0 | |
| 108 | Does the household currently have any of the following animals?(circle the answer) | | | |
|  | Oxen | | 1 | 0 |
|  | Cow | | 1 | 0 |
|  | Goat | | 1 | 0 |
|  | Sheep | | 1 | 0 |
|  | Chicken | | 1 | 0 |
| 109 | Does the household currently have any of the following items? (circle the answer) **(1. Yes 0. No)** | | | |
|  | Functioning radio | | 1 | 0 |
|  | Functioning television | | 1 | 0 |
|  | Functioning tape recorder/CD player | | 1 | 0 |
|  | Kerosene stove | | 1 | 0 |
|  | Telephone | | 1 | 0 |
|  | Electric stove? | | 1 | 0 |
|  | Sofa | | 1 | 0 |
|  | Bed | | 1 | 0 |
|  | Spring mattress | | 1 | 0 |
|  | Foam mattress | | 1 | 0 |
|  | Grass mattress | | 1 | 0 |
|  | Chair | | 1 | 0 |
|  | Table | | 1 | 0 |
|  | Bicycle | | 1 | 0 |
|  | Telephone | | 1 | 0 |

**Part two: Child characteristics (ask information from primary care givers)**

| **Serial number** | **Questions** | **Option** |
| --- | --- | --- |
| 201 | Sex of child | 1.Male 2.Female |
| 202 | Age of child | _____________ (years) |
| 203 | Is your child having soil eating habit? | 1. Yes 2. No |
| 204 | Did your child receive deworming drug in the last one year? | 1 Yes 2. No |
| 205 | Does your child have habit of hand washing before eating foods? | 1 Yes 2. No |
| 206 | Does your child have hands washing habit after a toilet? | 1 Yes 2. No |
| 207 | Does your child attend nursery school? | 2. Yes 2. No |
| 208 | Does your child always wear shoes? | 1. Yes 2. No |
| 209 | Where do you wash your child’s body? | 1. Home 2. River 3. Other (specify) _________ |
| 210 | Does your child have habit of moving on barefoot? | 1. Yes 2. No |

**Part three: Knowledge and practice of mothers or guardian related to transmission & prevention of STH (ask information from primary caregivers)**

| **Serial number** | **Questions** | **Option** |
| --- | --- | --- |
| 301 | Do you know about STH infection? | 1. Yes 2. No |
| 302 | Sources of information related to STH (more than one answer is possible) | 1. Health facility 2. Community health worker  3. Radio/TV, talk in the community  4. No information |
| 303 | How STH is transmitted? (don’t read option and circle the mentioned) | - Contaminated foods (1. Yes 0. No) - Contaminated water (1. Yes 0. No) - Not washing hands before cooking foods (1. Yes 0. No) - Not washing hands before eating foods (1. Yes 0. No) - Not washing hands after toilet (1. Yes 0. No) - Not washing hands before feeding children (1. Yes 0. No) - Soil eating habit of child (1. Yes 0. No) - Not washing fruits and vegetables before eating (1. Yes 0. No) - Untrimmed and long nail (1. Yes 0. No) - Eating raw foods (1. Yes 0. No) - Not using toilet (1. Yes 0. No) |
| 304 | How STH is prevented and do you practice?  (don’t read option and circle the mentioned) | - Food hygiene (1. Yes 0. No) - Having safe water (1. Yes 0. No) - Washing hands before cooking foods (1. Yes 0. No) - Washing hands before eating foods (1. Yes 0. No) - Washing hands after toilet (1. Yes 0. No) - Washing hands before feeding children (1. Yes 0. No) - Soil eating habit (1. Yes 0. No) - Washing fruits and vegetables before eating (1. Yes 0. No) - Having trimmed nail (1. Yes 0. No) - Not eating raw foods (1. Yes 0. No) - Using toilet (1. Yes 0. No) - Treating infected individuals (1. Yes 0. No) |

**Part four: Water, Sanitation and Hygiene (WASH) characteristics of households ask information from primary caregivers)**

| **Serial number** | **Questions** | **Option** |
| --- | --- | --- |
| 401 | What is your main source of drinking water? | 1.Private tap  2. Public tap  3. Well  4. Other (specify) _________ |
| 402 | Do you obtain sufficient amount of water for household consumption? | 1. Yes 2. No |
| 403 | Do you treat water before drinking? | 1.Yes  2. No (if no skip to q406) |
| 404 | How do you treat water? | 1. Boiling 2. Chemical (agar)  3. Both |
| 405 | How often do you treat water? | 1. Always 2. Usually  3. Sometimes 4. Never |
| 406 | How far is the water source from home? | ____ (minutes) |
| 407 | Do you have latrine? | 1.Yes 2.No (if no skip to q413) |
| 408 | Which type of latrine do you have? | 1. Simple pit 2. Ventilated improved pit latrine (VIP)  3. Pour flush 4. Other (specify) |
| 409 | Is the latrine clean? (Faeces or any dirt not observed on latrine floor) | 1.Yes 2.No |
| 410 | Functional hand washing station available around latrine? | 1.Yes 2.No |
| 411 | Do you wash your hand after toilet? | 1.Yes 2.No |
| 412 | Is there soap or ash at hand washing station? | 1.Yes 2.No |
| 413 | What is the reason for not having latrine? | 1. Have no place  2. No money  3. legal permission is difficult  4. Lack of knowledge  5. No |
| 414 | Where do you defecate? | 1.Open field  2. By payment in hotels 3. Other (specify)­_________ |
| 415 | Where do you dispose child’s faeces? | 1. Anywhere in the compound  2. In toilet  3. In garbage box  4. Other( specify) _________ |
| 416 | Do you wash your hands before cooking foods? | 1. Yes 2. No |
| 417 | Do you wash your hands after working? | 1. Yes 2. No |
| 418 | Do you wash raw fruits or vegetables? | 1. Yes 2. No |
| 419 | Do you wash your hands before eating foods? | 1.Yes 2.No |
| 420 | Do you wash your hands after cleaning your child? | 1.Yes 2.No |
