# Supplementary material for: Determinants of soil-transmitted helminth infections among pre-school-aged children in Gamo Gofa zone, Southern Ethiopia: A case-control study
Source: PLoS One. 2020 Dec 11;15(12):e0243836. doi: 10.1371/journal.pone.0243836 (PMC7732061; doi:10.1371/journal.pone.0243836)
Supplement: Attachment — Submitted filename: Review comments.docx [file pone.0243836.s003.docx]

**Determinants of soil-transmitted helminth infection among pre-school-age children in Gamo Gofa zone, Southern Ethiopia: a case-control study**

The authors present the findings on determinants of STH infection among PSAC in a study area in Southern Ethiopia. The study provides evidence on the association of STH and WASH factors among PSAC group of children. Therefore, this is a relevant study and of interest to STH control programs.

General comments:

The entire manuscript should be checked and proofread for grammatical and spelling corrections

Specific comments:

Abstract:

Include the cut-off for lower mean score for KP, line 47.

Introduction:

Generally well written.

Methods:

Avoid a one sentence paragraphs. Please provide more and explicit information on the study design. Lines 96-97.

The stated age group of 1-5 years is not the generally used PSAC age group, for comparability of the results, amend the analysis using the generally used PSAC age group.

State how STH infection was defined as an outcome variable, was it any STH positive result? Line 119.

How was the questionnaire administered? Paper or technology-based? State how the method used guaranteed data quality and integrity. Lines 124-126.

How were scores for KP created? Lines 145-146

Usually, multivariable logistic regression model is build following a univariable logistic regression and variable selection process. Was univariable logistic regression conducted? Perhaps a table summarizing this result is necessary. However, I have noted that a bivariate analysis was conducted using chi-square test, you don’t need to present its results. Just note that univariable logistic regression is different with chi-square test. Why was p<0.25 considered for the bivariable analysis?

State the ethical approval number if the study was reviewed and approved. State if assent was obtained from children. Why was written consent not obtained from the parents/household heads? Lines 152-155

Results:

Give a paragraph summarizing the infection prevalence by each individual species and any STTH among the participants.

Instead of running the logistic regression model on only any STH, considering doing this analysis on each STH species as well. This would inform on the WASH variation on individual species.

State in the methods how latrine cleanliness was defined, lines 176.

Tables 3 and 4: Instead of writing “1” write “reference”, since this refers to the reference category. Put table note explaining what “--” means. Separate the multivariable analysis on its own table. Delete chi-square test results and put the univariable logistic regression results instead. Give the definition of unsafe/safe water source. Where are the results of multivariable analysis in table 4?

Discussion:

Generally written well but should be improved after addressing the above concerns touching on methods and results.
