# Supplementary material for: Determinants of soil-transmitted helminth infections among pre-school-aged children in Gamo Gofa zone, Southern Ethiopia: A case-control study
Source: PLoS One. 2020 Dec 11;15(12):e0243836. doi: 10.1371/journal.pone.0243836 (PMC7732061; doi:10.1371/journal.pone.0243836)
Supplement: Attachment — Submitted filename: Review comments R1.docx [file pone.0243836.s005.docx]

**Determinants of soil-transmitted helminth infection among pre-school-age children in Gamo Gofa zone, Southern Ethiopia: a case-control study**

The authors have done a great job to address explicitly all my earlier comments. However, as I had stated earlier, some grammatical and sentence construction errors remain that need to be addressed. Perhaps, an independent copy editing need to be considered. Additionally, the authors need to make it clear whether they used P<0.25 (line 202) or P≤0.25 (line 209) for univariable analysis, make this consistent throughout the text. In table 3, indicate the word “Reference” on the comparison categories.
