# Supplementary material for: Determinants of soil-transmitted helminth infections among pre-school-aged children in Gamo Gofa zone, Southern Ethiopia: A case-control study
Source: PLoS One. 2020 Dec 11;15(12):e0243836. doi: 10.1371/journal.pone.0243836 (PMC7732061; doi:10.1371/journal.pone.0243836)
Supplement: Attachment — Submitted filename: Response to Reviewers.docx [file pone.0243836.s006.docx]

**Authors’ Responses**

Authors highly valued and appreciate the editor’s and reviewers’ comments. The manuscript has been modified accordingly.

**Response to Reviewer’s comments**

**Reviewer #1**

1. The authors have done a great job to address explicitly all my earlier comments. However, as I had stated earlier, some grammatical and sentence construction errors remain that need to be addressed. Perhaps, an independent copy editing need to be considered.

**Response:** Authors are grateful for the reviewer’s comments. With special attention given to grammatical and sentence construction errors, the entire manuscript has been checked and proofread for grammatical and spelling corrections.

2. Additionally, the authors need to make it clear whether they used P<0.25 (line 202) or P≤0.25 (line 209) for univariable analysis, make this consistent throughout the text.

**Response:** Agreed, thank you. Correction has been made. And please note that we used at P ≤0.25 to select potential variables for fitting into multi-varable model. We made correction on the revised version of the manuscript accordingly.

3. In Table 3, indicate the word “Reference” on the comparison categories.

**Response:** Agreed, correction has been made in the revised version.
