# Supplementary material for: Determinants of soil-transmitted helminth infections among pre-school-aged children in Gamo Gofa zone, Southern Ethiopia: A case-control study
Source: PLoS One. 2020 Dec 11;15(12):e0243836. doi: 10.1371/journal.pone.0243836 (PMC7732061; doi:10.1371/journal.pone.0243836)
Supplement: Attachment — Submitted filename: Response to Reviewers.docx [file pone.0243836.s007.docx]

**Authors’ Responses**

Authors are grateful for the editor’s comments, and the manuscript has been amended accordingly.

**Response to editors’ comments**

1. Table 3: Faeces or any dirt observed on latrine surface, in the Multi-variable analysis, AOR (95%CI) column provide the reference category.

**Response:** Agreed, correction has been made in the table at line 235.

2. Table 3. Educational status of caregivers, provide the Univariable analysis, COR (95%CI) for the categories

**•** Can read and write

• Elementary

• Secondary

**Response:** Agreed, correction has been made in the table at line 235.
